# Effect of vitamin D deficiency on postorthodontic relapse: An animal study

**DOI:** 10.1002/cre2.765

**Published:** 2023-07-13

**Authors:** Asmaa M. Khamees, Dheaa H. Al Groosh

**Affiliations:** ^1^ Department of Orthodontics, College of Dentistry University of Baghdad Baghdad Iraq

**Keywords:** orthodontic, relapse, vitamin D

## Abstract

**Objectives:**

This study aims to evaluate the effect of vitamin D deficiency (VDD) on orthodontic tooth movement (OTM), retention, and relapse and to assess the effect of systemic administration of vitamin D (VD) in a rat model.

**Materials and Methods:**

A total of 32 male Wistar rats were divided into two groups, a control group of 11 rats and an experimental group of 21 rats with VDD, after enhancement using a custom diet. Of the VDD group, 11 rats were supplemented with systemic vitamin D3 and categorized as vitamin (VD supplement [VDS]) groups. The VDS group received 40,000 IU/kg via intramuscular injection on Days 1 and 15 of the orthodontic treatment period. A modified orthodontic appliance was fitted to apply 0.5 N of force to move the maxillary right first molars mesially for 14 days, followed by retention and relapse periods for 7 days. Space created during OTM was measured and reassessed after the retention and relapse periods. The relapse ratio was estimated, and histomorphometric analysis was performed to assess the number of osteoblasts, osteoclast bone cells, and bone area.

**Results:**

A significant increase in the relapse ratio and a reduction in osteoblast cells and bone area were observed in the VDD group. By contrast, the amount of tooth movement was significantly higher together with osteoblast cells and bone marrow in VDS with a significant reduction in relapse ratio.

**Conclusion:**

VDD was associated with a significant reduction in osteoblast cell count and total bone area in addition to a significant increase in relapse ratio. Routine screening of VD may be beneficial before commencing orthodontic treatment.

## 1 INTRODUCTION

Relapse in orthodontics is considered one of the undesirable side effects of orthodontic treatment (Littlewood et al., 2016). Many studies found that the prevalence of relapse after orthodontic treatment ranged from 30% to 50% of the satisfactory alignment of orthodontic patients after 10 years, and only 10% of the treated cases retained an acceptable alignment after 20 years (Yu et al., 2013). Numerous factors might contribute to orthodontic relapses, such as periodontal, muscular, anatomical, sex, occlusal, and orthodontic treatment mechanics‐related factors (Ben Mohimd et al., 2018), in addition to other factors, including habits, neuromuscular imbalance, and continuous craniofacial growth (Littlewood et al., 2016). Substantial research has been conducted to reduce the relapse ratio using pharmaceutical or nonpharmaceutical approaches.

The biological mechanisms of orthodontic tooth movement (OTM) and relapse are similar, and they resemble tissue responses when initial force is applied to a tooth, that is, the pressure side exhibits an increase in the activity of osteoclasts (McManus et al., 2014). However, OTM exhibits a reduction in alveolar bone density, whereas relapse shows an increase in density (Maleeh et al., 2016).

Currently, vitamin D deficiency (VDD) is considered a major public health problem that occurs in almost all age groups. Different factors (e.g., racial factors, which include skin pigmentation, and cultural factors, such as age, gender, clothing, limited skin exposure to sunlight, and the use of sunscreen protectors) may contribute to VDD (Cashman et al., 2012; Forrest & Stuhldreher, 2011; Thuesen et al., 2012). Other factors, including time of day, season of the year, latitude, and altitude, may influence VD3 production (Holick, 2006; Yetley et al., 2009).

Epidemiologic studies have shown that VDD is associated with lower bone mineral density and fractures. It increases serum parathyroid hormones, which may successively result in bone loss and mineralization defects. These outcomes can be avoided with modest doses of vitamin D (VD) and calcium supplements (Hatun et al., 2011; Munns et al., 2016). VD was found to be associated with bone homeostasis, osteoclastic resorption activity, and an increase in the rate of intestinal calcium and phosphate absorption (Holick, 2007). The main function of VD in the body is to maintain serum calcium and phosphate concentrations and hence is connected with important physiological functions, such as normal mineralization of bone, muscle contraction, nerve conduction, and prevention of hypocalcemic tetany (Yetley et al., 2009).

In orthodontics, VD screening is not a routine process, and the chance of having those patients scheduled for treatment is high. To the best of the authors' knowledge, information about orthodontic treatment and relapse in patients with VDD is limited. This study was designed to evaluate the effect of VDD on OTM, retention, and relapse, and to assess the effect of the systemic administration of VD in a rat model.

## 2 MATERIALS AND METHODS

The study was approved by the scientific research and ethics committee (issue number 177, date 16/1/2020).

### 2.1 Sample preparation

A total of 32 male Wistar rats of 220–300 g in weight with an age range of 8–10 weeks were included in the study. Animals were housed in the Center of Cancer and Medical Genetics Research (CCMGR), where they were acclimated for approximately 7 days before the experiments. Rats were subjected to 12/12 h of dark/light cycles under a constant temperature of 21°C ± 2°C and relative humidity of 50% ± 10%. Water was available ad libitum; additionally, food was a standard laboratory pellet (Al‐Harbi et al., 2017).

Each animal was weighed and labeled (coloring the tail) and kept in groups of 3–4 rats in each cage. Blood samples were taken from all rats by directly drawing from the heart using a sterile disposable syringe of 5 mL (Medical Devices) after anesthetizing the animals with chloroform (Alpha Chemika). Blood samples were directly stored in vacuum blood collection tubes (gel and clot activator tube glass, 6 mL; IVD), allowed to clot for 30 min and then centrifuged at 3000 rpm for 10 min according to the manufacturer's instructions (MyBioSource). The serum was then separated in a 2 mL frozen serum tube (Eppendorf Tubes) and stored in a −20°C refrigerator (Instruction Guide; MyBioSource).

A VD antibody protein Enzyme‐Linked Immunosorbent Assay Kit (MyBioSource) was used to assess VD levels, and serum calcium was calculated using a photometric test (Human, Diagnostics Calcium Kit). According to serum VD level, rats were divided into two groups as follows:

1. *Control group*: This group consisted of 11 rats with normal VD (>15–20 ng/mL) (Stavenuiter et al., 2015) that were given standard rat pellets.
2. *Experimental group (VDD)*: This group consisted of 21 rats with normal or borderline normal levels of VD (<15 ng/mL). The rats were subjected to a custom diet deficient in VD (casein‐free VD; Bio‐Serv) for 21 days (Hokugo et al., 2010; Stavenuiter et al., 2015).

**Figure 1:**
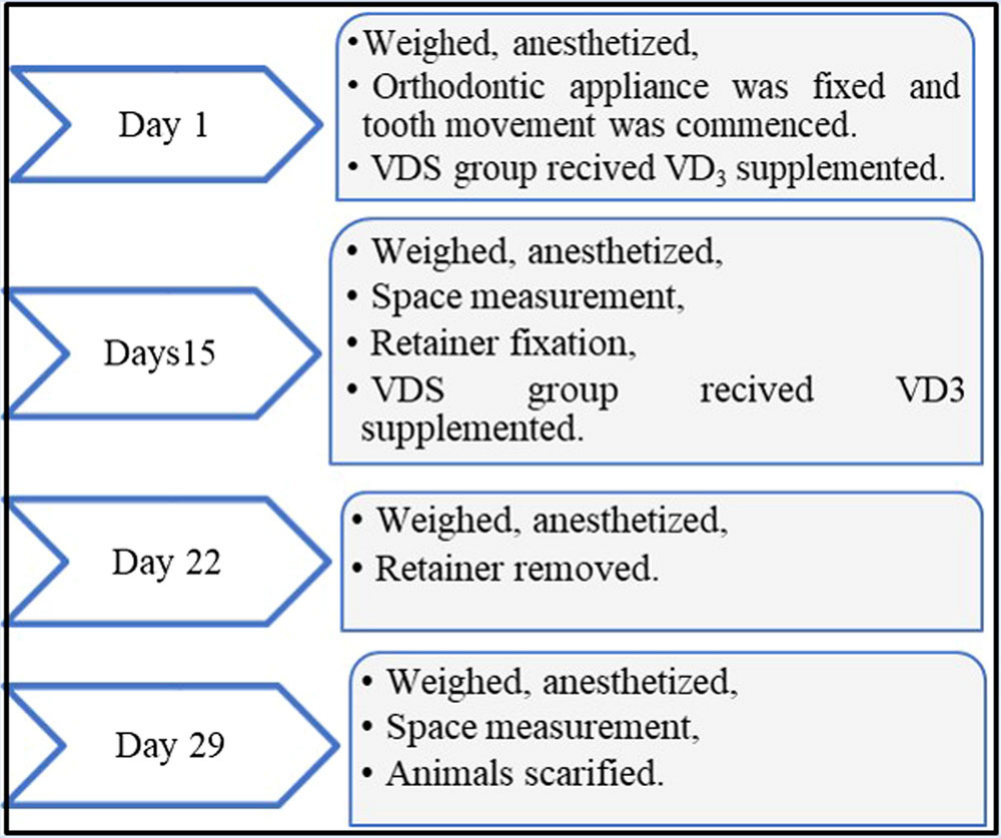
Chronological phases of orthodontic intervention. VD, vitamin D; VDS, VD supplement.

After 21 days, rats were weighed, and blood samples were collected to confirm VD and Ca levels. In the experimental group, the VD level was <5 ng/mL. The experimental group was then divided into two subgroups:

1. The VDD group consisted of 10 rats that did not receive any intervention.
2. The VDD with VD supplement group (VDS) consisted of 11 rats that received two doses of 40,000 IU/kg and cholecalciferol 300,000 IU/1 mL (Dibase; Abiogen) (Derakhshanian et al., 2017). All animals underwent orthodontic treatment with a fixed appliance for 14 days, followed by retention and relapse periods of 7 days each (Figure 1).

### 2.2 Insertion of orthodontic appliance

According to the animal housing guidelines in CCMGR, each rat was weighed and then anesthetized using intramuscular injection of ketamine (87 mg/kg) (ketamine 10%; Alfasan) and xylazine muscle relaxant (10 mg/kg) (XYL‐M2 injectable solution 25 mL; VMD) at a ratio of 2:1 in thigh muscle (Alnajar & Al Groosh, 2020). This process was repeated during the OTM, retention, and relapse phases. To limit the influence of interanimal variation in response to metabolic stimuli, unilateral orthodontic appliances were bonded, and the untreated contralateral side served as a control (Verna, 2000) (Figure 1).

**Figure 2:**
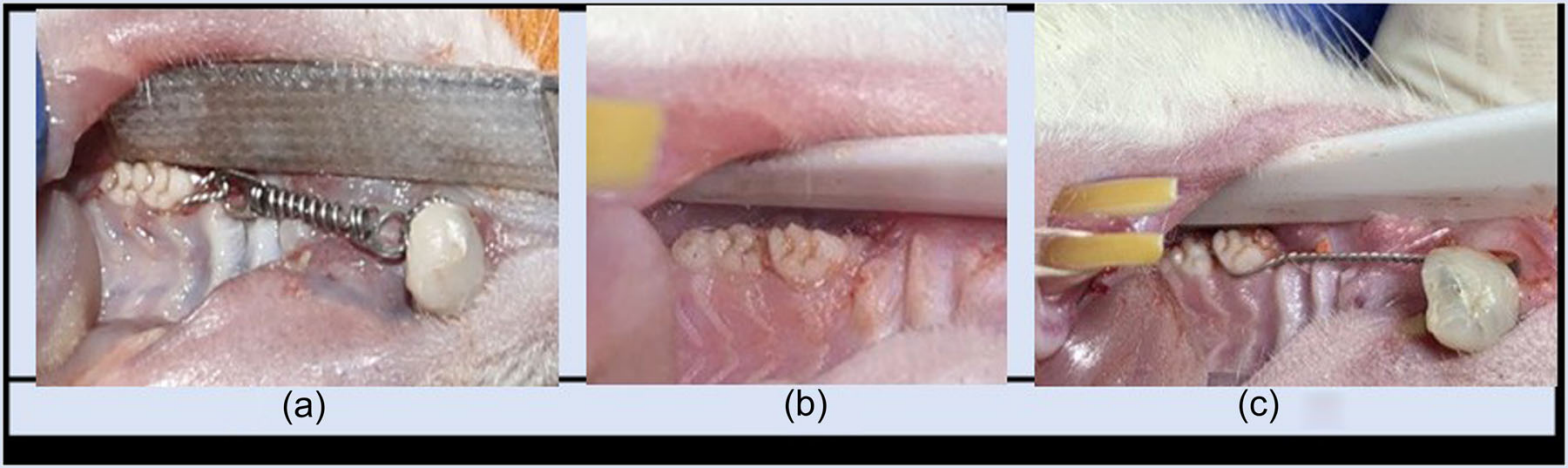
Orthodontic intervention. (a) Orthodontic fixed appliance. (b) space created by orthodontic appliances. (c) Retainer fixation.

According to Yadav et al. (2015) and Alnajar and Al Groosh (2020), a modified orthodontic appliance was fixed on the right side of the upper arch using an orthodontic ligature wire (0.010″, Truforce stainless steel; Ortho Technology), which was inserted interdentally between the first and second right maxillary molars, tightly ligated and wined around the first maxillary molar, and access of 4–5 mm was left as a hook for the attachment of a nickel–titanium closed‐coil spring (nickel–titanium closed‐coil spring, Dentaurum, Rematitan® LITE tension spring, Dentaurum). The other end of the spring was attached to the maxillary central incisors using a ligature wire (0.012 Kobayashi; Klardent) ligated and fixed within grooves prepared (to prevent dislodgement of the wire) on the facial, distal, and mesial surfaces of the maxillary incisors. The labial surface and grooves were etched with acid etch gel (37% Microdont) for approximately 1 min, rinsed with distal water, and dried until a white chalky area appeared. Then, the delivered force was adjusted to 0.5 N using a digital hand‐held force gauge (Sr‐1 kg Gray Digital Hanging Scale; American Weigh Scales) to move the first molar mesially. Afterward, the anterior ligature wire was fixed using composite material, bonding adhesive (Adper, single bond 2, 3M ESPE), and composite filling materials (Universal Restoration; Z350 XT, Filtek 3M ESPE), according to the manufacturer's instructions (Figure 2a) (Alnajar & Al Groosh, 2020).

Interproximal space between the distal surface of the first molar and the mesial surface of the second molar was measured using an interproximal Vernier caliper (feeler gauge, ROSHTOO 80). All measurements were performed twice by one operator (Franzen et al., 2011).

For the VDS group, VD3 (cholecalciferol 300,000 IU/1 mL) was administered at 40,000 IU/kg in the left thigh muscle on the first day and at the end of the orthodontic treatment (Derakhshanian et al., 2017). On Day 15, the animals were weighed and anesthetized, and the interproximal space was measured (Figure 2b). The orthodontic appliances were removed, and a retainer was fitted using a prestretched ligature wire (Ortho Technology) extended from the first molar to the incisors, keeping the level of force as low as possible to avoid continuous mesial movement or avulsion of the tooth. The retainer was left for 7 days (Li et al., 2016; Öztürk & Gül Amuk, 2021) (Figure 2c).

After the retention period, the animals were anesthetized, and the retainer was removed. The interproximal space between the first and second molars was kept without changes through the retention period (Franzen et al., 2011). After 7 days, that is, the relapse period, an overdose of ketamine and xylazine (ratio 2:1) was given, and the final interproximal space was measured.

### 2.3 Relapse ratio measurement

The interdental space before and after OTM, after the retention phase, and after the relapse period was measured between the maxillary right first molar and second molar using an interproximal Vernier caliper (Feeler gauge). The rate of relapse was assessed using the following equation:

Amountofrelapse(SD)=(D1−D2)andtherelapseratio(RR)=/D1×100%,

where *D*1 = space after 14 days of orthodontic force and *D*2 = space after 7 days of relapse.

The interproximal space difference after tooth movement and after retention was neglected (<0.01 mm).

### 2.4 Histological sample preparation

The rats were killed under an overdose of anesthesia, and the maxilla was separated and placed in 10% natural buffered formalin solution (formaldehyde; Scharlab S.L.) for tissue fixation using a labeled plastic container (according to the rat number in the correlated group) for 24 h. After fixation, the specimens were washed thoroughly with running water and rinsed in 10% EDTA solution (BDH Chemicals Ltd.) with a pH of 7 for 21 days, which was changed every alternative day, to decalcify the teeth and the bone (Alnajar & Al Groosh, 2020; Plut et al., 2015; Sarsfield, 2000). After decalcification, each maxilla was dissected into two halves, and each half involved three maxillary molar teeth (Adachi et al., 1994; Al‐Duliamy, et al., 2015). The sample was rinsed with distilled water and then dehydrated using successive concentrations of alcohol (González‐Chávez et al., 2013). The sample, the maxilla, was then poured with paraffin wax (Leica Biosystems) and kept in an orientation that allowed longitudinal sections of teeth with periodontium to be obtained.

### 2.5 Histological analysis

#### 2.5.1 Histological slide preparation

Two 5‐µm‐thick slices were obtained through the mesial root of the maxillary first molar, including the pressure and tension sides. The section was mounted on a clean glass slide (Surgipath, Snowcoat, Pearl Slides; Leica), and hematoxylin and eosin staining (HE; Leica Biosystems) was performed in accordance with Ihcworld H&E staining method and protocol (Al‐Duliamy et al., 2015).

Briefly, the histological sections were deparaffinized with xylene and dehydrated in descending alcohol concentrations. First, the section was stained with Harris hematoxylin stain for 7–10 min and washed in tap water for 1–5 min to remove the excess stain. Then, the slide section was stained with eosin for 1–2 min and dehydrated in 95% and 100% alcohol (Diamon D, Alcool Ethylique) for 2–3 min successively and finally placed in xylene. Afterward, a cover slip was fixed on the stained tissue sections using Canada balsa (Dako). Histological examination was performed under a light microscope (OPTIKA, Microscopes) with objective lenses of ×4, ×10, and ×40 to evaluate the histological changes in the groups. The histological sections were photographed using a photomicroscope (Olympus).

Photographs were taken for the mesial root of the upper first molar for the pressure side and the tension side. The sections were assessed by examining four microscopic fields to count the number of involved cells and to assess the surrounding bone. For interexaminer calibration, two randomly selected sections were examined by the same histopathologist in a blind test (Hudson et al., 2012). The mean of the readings of the four microscopic fields was calculated and used in statistical analysis.

#### 2.5.2 Osteoblast and osteoclast bone cells calculation

The microscopic field of the root and surrounding bone were divided into four fields. Osteoclast cells, multinucleated large cells located in Howships lacuna adjacent to the root surface, osteoblast cells, and cuboid cells with large nuclei located on the surface of the osteoid or bone were counted in the pressure and tension sides of the mesial root of the maxillary right first molar using a light microscope with a ×40 magnification lens with an aid of a graduated eyepiece. The average number of cells in all fields was taken to represent the cell count in the pressure or tension sides of the root (Haq et al., 2016; Ong et al., 2000).

#### 2.5.3 Bone area

**Figure 3:**
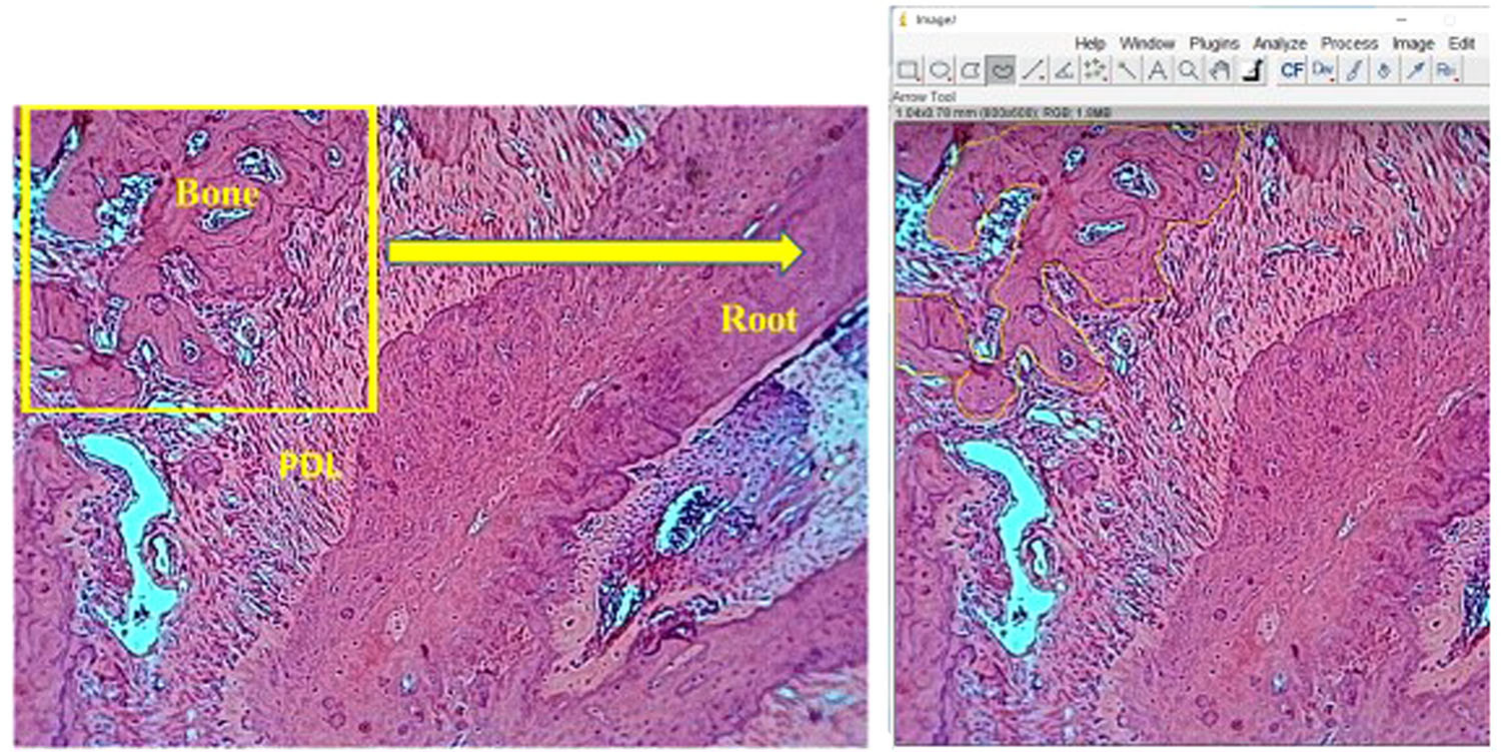
ImageJ software for bone area measurements. The yellow box represents the way of using the ImageJ processing program. PDL, periodontal ligament.

Measurement of the total bone area in pressure and tension sides around the mesial root of the upper right first molar (Plut et al., 2015) was performed by the ImageJ processing program (ImageJ.exe), developed at the National Institutes of Health (Razouki & Ghani, 2016; Schneider et al., 2012). Using a camera attached to the microscope at a magnification power of ×10, two microscopic pictures were taken (Figure 3). The selected bone area was measured in pixels, then converted to mm to represent the total bone area around the mesial root (Rha et al., 2015).

### 2.6 Statistical analysis

The collected data were statistically evaluated by using SPSS version 25. The Shapiro‐Wilk test was used to test the normality of the data distribution. Accordingly, Kruskal Wallis test, pairwise tests, and Wilcoxon signed‐rank tests were used for comparisons of rat weight, VD, calcium, tooth movement, and bone cell counts. Analysis of variance and Games–Howell tests were used to compare the bone area.

## 3 RESULTS

### 3.1 Laboratory investigation during treatment stages

**Figure 4:**
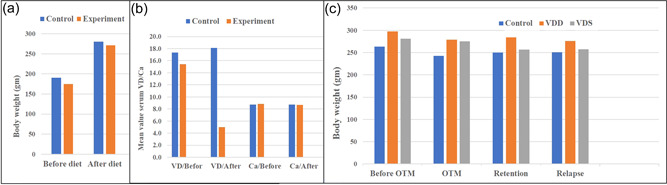
The mean values of the following: (a) Rat body weight before and after applying the custom diet. (b) Vitamin D (VD) and calcium levels before and after applying the custom diet. (c) Rats body weight during orthodontic phases. OTM, orthodontic tooth movement; VDD, vitamin D deficiency; VDS, vitamin D supplement.

Rat body weight, VD, and serum calcium levels were evaluated before and after diet application. Figure 4a,b shows a nonsignificant difference in rat weight and calcium levels regardless of diet type throughout the experiment. However, VD levels revealed a significant difference between groups, where the serum VD level decreased significantly after the custom diet in the experimental groups (Figure 4b).

### 3.2 Body weight during orthodontic phases

A nonsignificant change in rat weight was observed between the three groups during the orthodontic phases, that is, before orthodontic treatments, OTM, during retention, and after relapse (Figure 4c).

### 3.3 OTM

**Figure 5:**
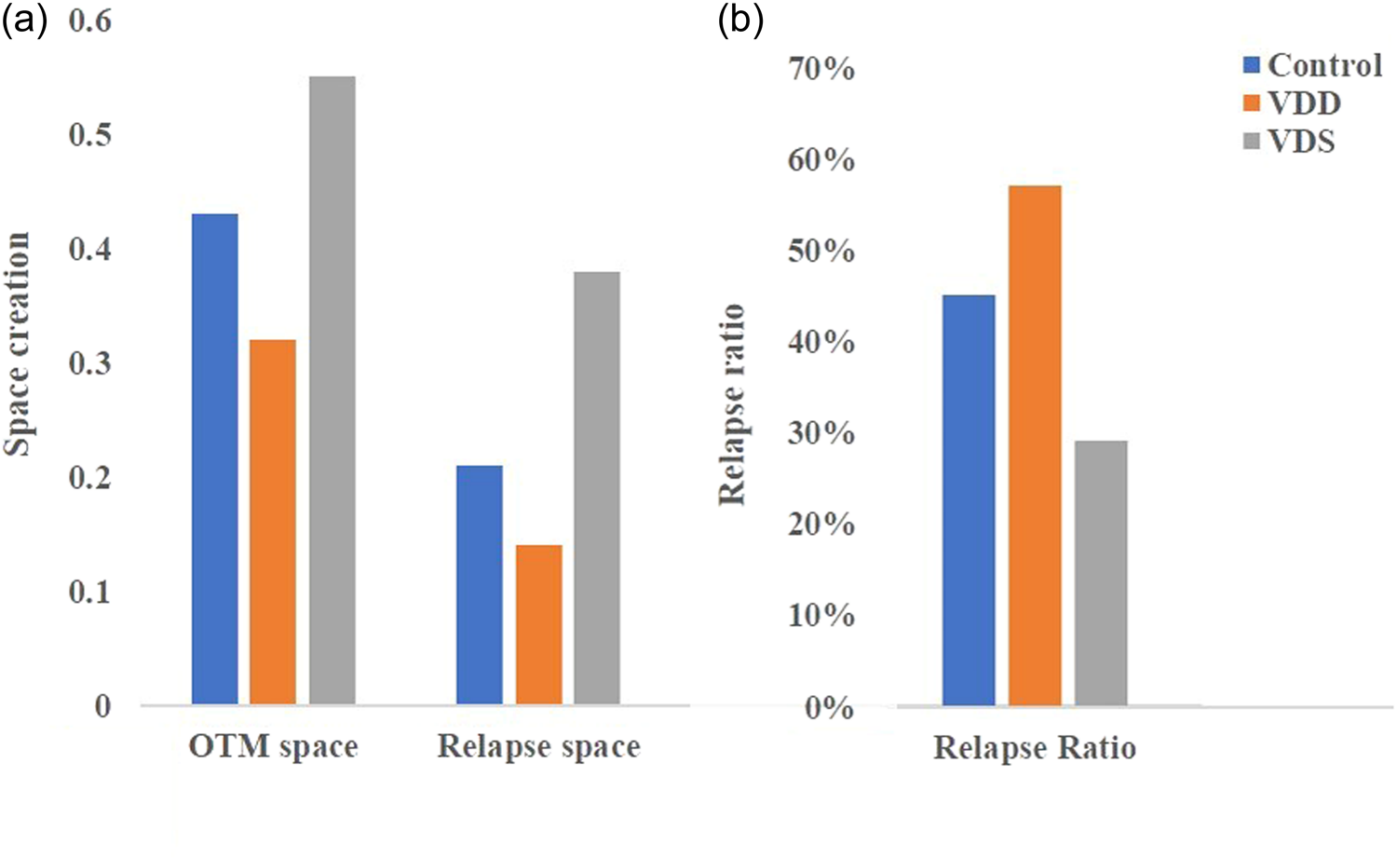
The mean values of the following: (a) OTM space and relapse space and (b) relapse ratio. OTM, orthodontic tooth movement; VDD, vitamin D deficiency; VDS, vitamin D supplement.

Figure 5a shows that the mean value of spaces created through OTM and that remained after relapse was significantly higher in the VDS group than in the VDD group. Moreover, the relapse ratio was significantly higher in the VDD group and lower in the VDS group (Figure 5b).

### 3.4 Histomorphometric analysis

#### 3.4.1 Osteoblasts and osteoclast cells in the pressure side

**Figure 6:**
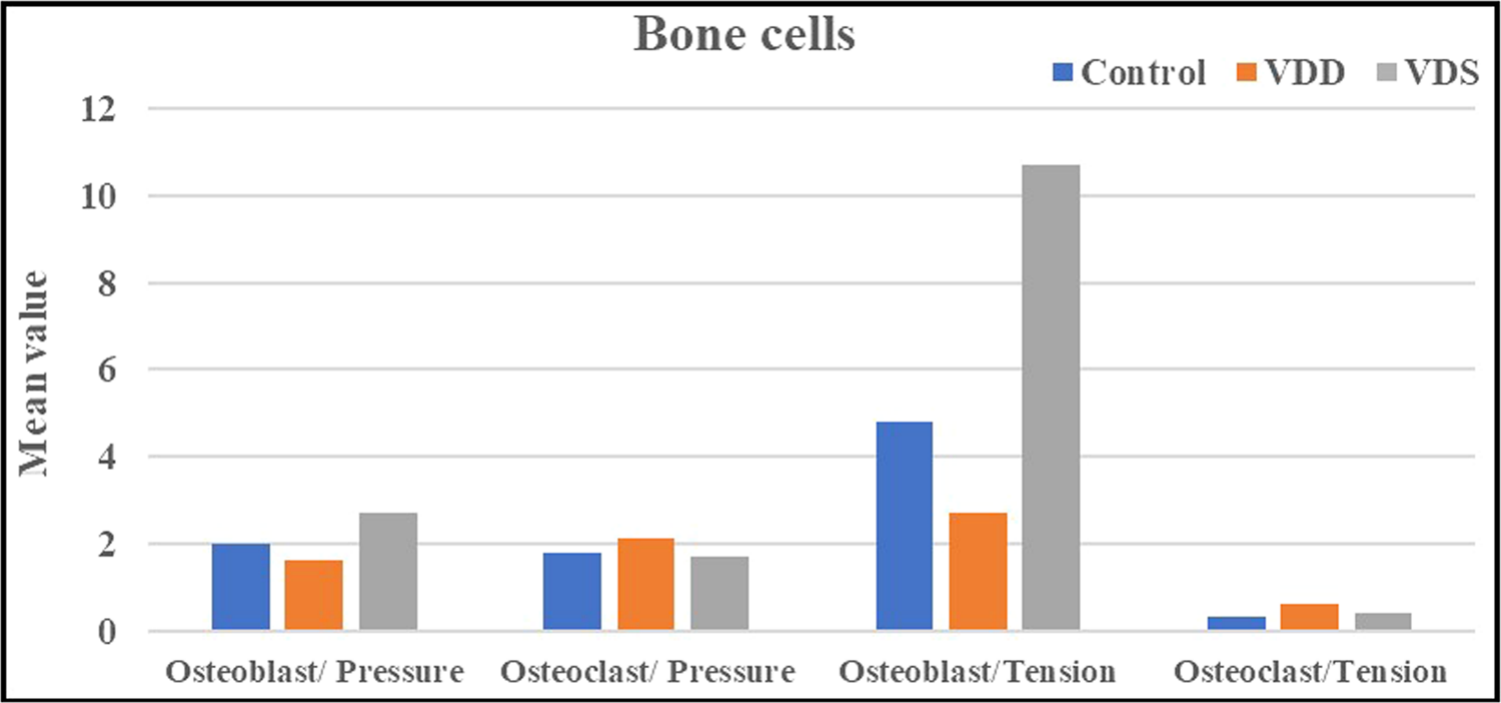
The mean values of the osteoblast and osteoclast cells in the pressure and tension sides. VDD, vitamin D deficiency; VDS, vitamin D supplement.

Data revealed a nonsignificant difference among the groups regarding the number of osteoclast cells. However, the osteoblast cell count showed a significant increase in the VDS group compared with the VDD group (Figure 6).

#### 3.4.2 Osteoblasts and osteoclasts cells in tension side

Similar to the pressure side, a nonsignificant difference in the osteoclast cell count was found on the tension side among the groups. However, the osteoblast cell counts showed a significant increase in the VDS group compared with the control or VDD groups (Figure 6).

#### 3.4.3 Bone area

**Figure 7:**
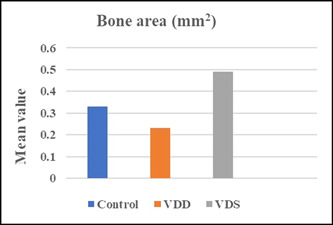
The mean value of the bone area around the mesial root of the upper maxillary first molar. VDD, vitamin D deficiency; VDS, vitamin D supplement.

**Figure 8:**
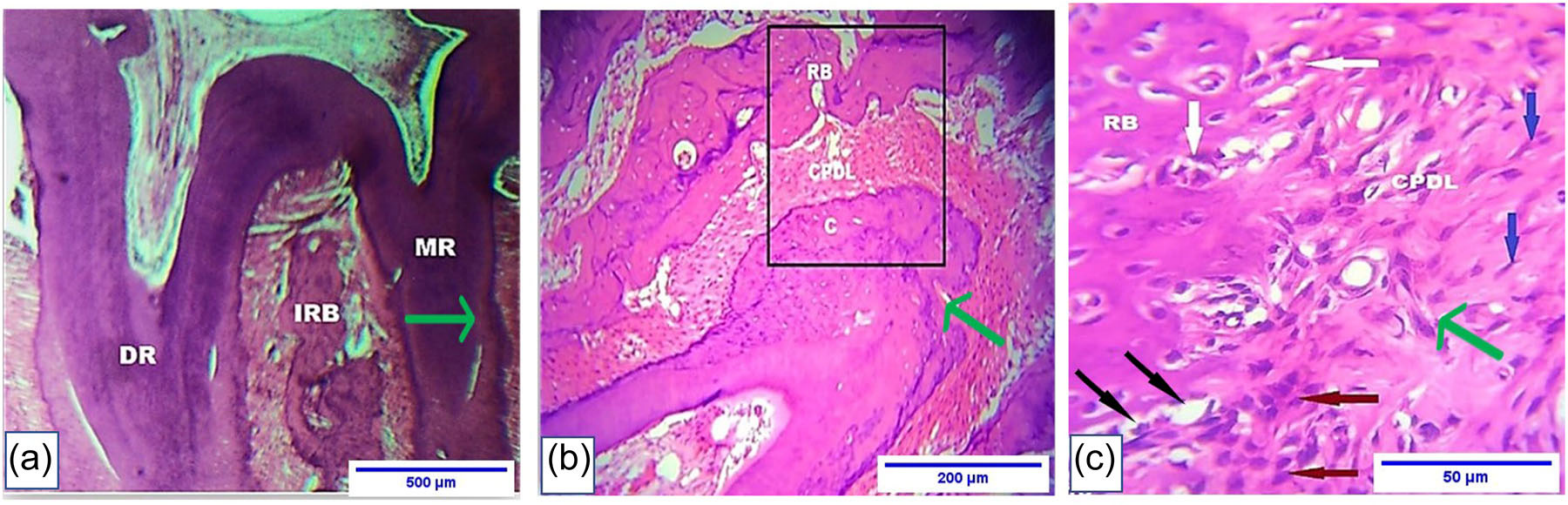
Microphotograph views of postorthodontic relapse at (29 days) for the upper maxillary first molar of rat that illustrate the following: (a) mesial root (MR), distal root (DR), and interradicular bone (IRB). H&E, ×4. (b) Compressed periodontal ligament (CPDL) adjacent to mesial root, bone resorption (RB), and cementum (C). H&E, ×10. (c) CPDL, RB, osteoclast (white arrows), osteoblast (black arrows), fibroblast (blue arrows), and inflammatory cells (brown arrows). The green arrow represents the direction of OTM, and the relapse represents the opposite direction. H&E, hematoxylin and eosin; OTM, orthodontic tooth movement.

**Figure 9:**
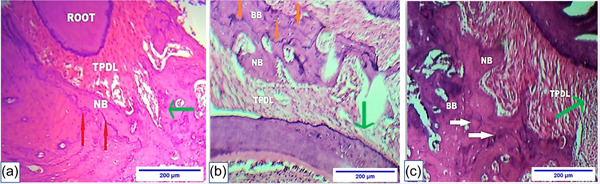
Microphotograph views of postorthodontic relapse at the end of the study for the upper maxillary first molar (cross section) of rat in the tension side of the periodontal ligament (TPDL) that illustrate bone deposition area. (a) Control group: reversal line (orange arrows), a demarked line separated basal bone (BB) and new bone (NB). (b) VDD group: reversal line (orange arrows) separated BB and NB. (c) VDS group: reversal line (white arrows) separated BB and NB. H&E, ×10. The green arrow represents the direction of OTM, and the relapse represents the opposite direction. H&E, hematoxylin and eosin; OTM, orthodontic tooth movement; VDS, vitamin D supplement.

Data showed a significant increase in the total bone area around the mesial root of the upper first molar in the VDS group. Additionally, a significant decrease in the total bone area was observed in the VDD group (Figures 7, 8, 9).

## 4 DISCUSSION

VDD is considered a major health problem in all ages, and its prevalence varies in different countries, ranging from 70% in the Caucasian population to 45%–100% in Asia (Hossein‐nezhad & Holick, 2012). VDD is associated with several bone‐related issues, that is, low bone mineral density and susceptibility to fracture (Hatun et al., 2011); these problems may have an effect on bone activity, which is a prime factor in orthodontics. This was true for tooth movement and retention after orthodontic treatment (Proffit et al., 2019). However, these bone anomalies may be avoided by modest doses of VD and calcium (Hatun et al., 2011; Munns et al., 2016). One of the most important concerns in orthodontics is the retention of the teeth after orthodontic treatment (Proffit et al., 2019). Previous research used local applications of biological agents, such as statins, strontium, bisphosphonates, tetracycline, raloxifene, and calcitonin, to decrease the amount of relapse (Al‐Duliamy et al., 2015; Alnajar & Al Groosh, 2020; Azami et al., 2020; Hudson et al., 2012; Veginadu et al., 2020; Vieira et al., 2019).

However, local applications have some drawbacks, such as the short half‐life of the agents, which requires multiple applications, and health‐related issues associated with prolonged administration (Arqub et al., 2021). Hence, in the current study, a systemic administration of VD was given with an optimum dose of 40,000 IU/kg (Tablas et al., 2018; Derakhshanian et al., 2017), and results show that 40,000 IU/kg elevated serum VD with no toxic effect. Repeated administration was given after 14 days dependent on the half‐life of serum 25D (Giangreco et al., 2015).

In the current study, VDD was induced in the experimental rat model using a VD‐free custom diet (Bio‐Serv) for 21 days. Results showed a significant reduction in serum VD compared with the control group, which agreed with Hokugo et al. (2010) and Stavenuiter et al. (2015), without affecting rat weight (Al‐Harbi et al., 2017; Ferreira et al., 2019; Hadjadj et al., 2019), or serum calcium levels (Hokugo et al., 2010; Tablas et al., 2018).

The results showed a significant increase in the amount of OTM in VDS compared with VDD and a nonsignificant difference among the other groups. This result could be due to 1,25‐dihydroxyvitamin D, which stimulates the expression of osteoblast signature genes (Atkins et al., 2007) via the VD receptor, which increases the expression of receptor activator of nuclear factor kappa (RANK) on the osteoclast progenitor cell surface. The binding of RANK to receptor activator of nuclear factor kappa‐B ligand activates and stimulates osteoclastogenesis and bone resorption and results in an increase in the amount of OTM. This result was in accordance with Takeda et al. (1999) and Lutter et al. (2016). Moreover, Collins and Sinclair (1988) and Kale et al. (2004) reported that local administration of VD accelerated tooth movement through the regulation of bone resorption and deposition processes.

The results agreed with many laboratory studies that reported that VDD may experience a slower rate of tooth movement (Boyce & Weisbrode, 1985; Kale et al., 2004; Kawakami & Takano‐Yamamoto, 2004).

Regarding the relapse rate, the current study found a significant reduction in the VDS group compared with the control and VDD groups. This result is probably due to the anabolic activity of VD on bone tissue, which increases the number of osteoblasts and bone minerals (Shevde et al., 2002). This finding was in accordance with the significant increase in the number of osteoblast cells and total bone area in the VDS group in relation to the other groups. The increase in mature osteoblast cells may be associated with the production of a new protein‐rich bone matrix that subsequently mineralized into new bone (Neag et al., 2021).

Results showed no significant difference in the number of osteoclast cells. This result could be due to the force‐related activity, where the number of osteoclasts on the compression sides appeared higher after 7 days of force application (Li et al., 2016); however, during force‐induced reversal action, that is, from bone resorption to bone formation, the number of osteoclast cells was reduced due to apoptosis (Kobayashi et al., 2000).

In conclusion, VDD was associated with a significant reduction in osteoblast cell count and total bone area in addition to a significant increase in relapse ratio. However, systemic administration of two doses of VD (40,000 IU/kg) resulted in an increase in tooth movement with a significant increase in osteoblast cells and total bone area, which may contribute to a significant reduction in the relapse ratio. Routine VD screening is advised as a protocol for patients planning for orthodontic treatment.

## AUTHOR CONTRIBUTIONS

**Asmaa M. Khamees**: Data curation, roles/writing—original draft, funding, investigation, software, formal analysis, visualization, and validation. **Dheaa H. Al‐Groosh**: Supervision, conceptualization, resources, writing—review and editing, funding, project administration, methodology, visualization, and validation.

## CONFLICT OF INTEREST STATEMENT

The authors declare no conflict of interest.

## Data Availability

Data are subject to third‐party restrictions.
